# P-717. Genetic Analysis of A(H3N2) Influenza Viruses and Associations with Host Characteristics among Kaiser Permanente Northern California Adults during the 2018-2019 Season

**DOI:** 10.1093/ofid/ofae631.913

**Published:** 2025-01-29

**Authors:** Ombeline Jollivet, Sandra S Chaves, Christophe Carré, Chao-Yang Pan, Hugo Guevara, Debra Wadford, Nicola P Klein, Amber Hsiao, Kamela Ng, Ruvim Izikson, Heidi Kabler, William T Harvey, Laurence Josset, Clotilde El Guerche-Séblain

**Affiliations:** Sanofi, Lyon, Rhone-Alpes, France; Sanofi Pasteur, Lyon, Languedoc-Roussillon, France; Sanofi, Lyon, Rhone-Alpes, France; California Department of Public Health, Center for Laboratory Sciences, Viral and Rickettsial Disease Laboratory, Richmond, California; California Department of Public Health, Center for Laboratory Sciences, Viral and Rickettsial Disease Laboratory, Richmond, California; California Department of Public Health, Richmond, CA; Division of Research Kaiser Permanente Vaccine Study Center, Oakland, California; Division of Research Kaiser Permanente Vaccine Study Center, Oakland, California; Sanofi, Lyon, Rhone-Alpes, France; Sanofi, Lyon, Rhone-Alpes, France; Sanofi Pasteur, Lyon, Languedoc-Roussillon, France; The Roslin Institute, University of Edinburgh, Edinburgh, Scotland, United Kingdom; Centre International de Recherche en Infectiologie, Univ Lyon, Inserm, U1111, Université Claude Bernard Lyon 1, CNRS, UMR5308, ENS de Lyon, Team Virpath, Lyon, Rhone-Alpes, France; Sanofi, Lyon, Rhone-Alpes, France

## Abstract

**Background:**

Influenza A(H3N2) viruses exhibit significant genetic diversity and mutations may escape acquired host immunity, potentially triggering infections among vaccinees. This study aims to explore associations between genetic changes in A(H3N2) viruses and vaccinal status of their adult hosts vaccinated with quadrivalent attenuated standard dose (SD-IIV4) vaccine, with recombinant (RIV4) vaccine, or not vaccinated, alongside exploration of associations with other host demographics.

Overview of virus and host populations characteristics.

**Figure:**
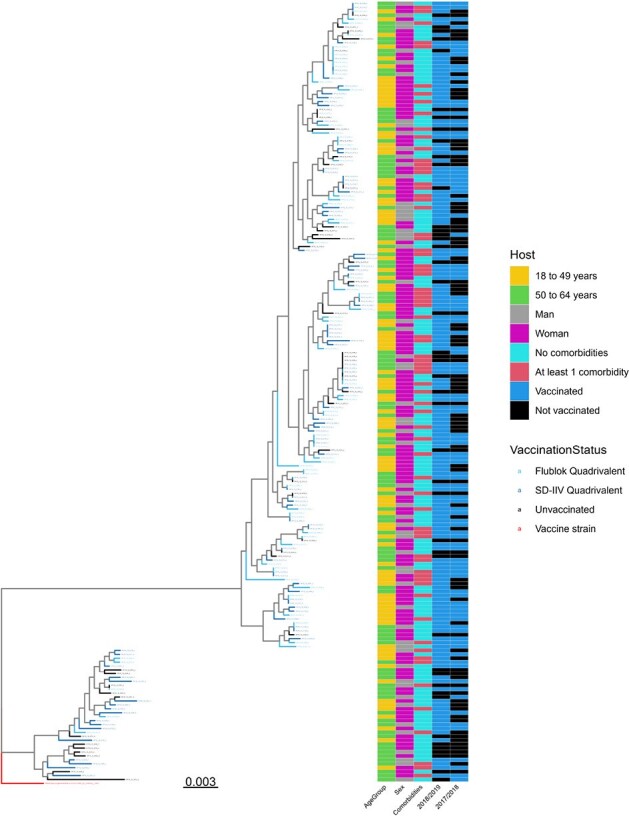

On the left part is shown the phylogenetic tree based on HA nucleotide sequences. On the right is displayed the clinical characteristics of the associated host population: from left to right, the columns represent age, sex, any comorbidities (asthma, diabetes, COPD, CHD), vaccination status in 2018/2019 season and vaccination status in the season just before, 2017/2018 season.

**Methods:**

We conducted whole genome sequencing of 192 A(H3N2) viruses collected during the 2018/2019 season from Kaiser Permanente Northern California members aged 18-64 years vaccinated with either SD-IIV4 (n=89) or RIV4 (n=62) or unvaccinated (n=41) who developed a mild influenza disease. Additional host variables included age, sex, underlying medical conditions and previous season (2017/2018) vaccination status.

Phylogenetic analyses were performed at nucleotide and amino acid level. Associations between specific mutations of hemagglutinin (HA) and neuraminidase (NA) and vaccine status were assessed by a) known antigenic drift mutations and b) agnostically, considering all mutations. Various clustering analyses were performed, including Multiple Component Analysis, Discriminant Analysis of Principal Components, and K-means.

Observed number of amino acid distinct patterns by the vaccination status.

**Figure:**
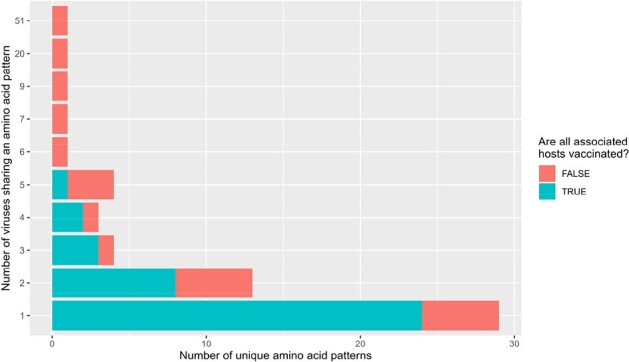

Blue represents when all viruses sharing a similar amino acid pattern were identified in vaccinated hosts, and red color when not, i.e. both the unique patterns were found for viruses detected in vaccinated and unvaccinated hosts. A distinct pattern is defined here as a - unique - set of hemagglutinin and neuraminidase amino acids.

**Results:**

Our sample set reflects the mismatch between circulating viruses and 2018/2019 vaccine composition (Figure 1). The observed viral genetic diversity among vaccinated individuals was higher than those who were unvaccinated (Figure 2): among 58 observed unique amino acid combinations, 64% were found solely in those vaccinated. The vaccine type administered was rarely associated with HA mutations known to trigger a drift: 1 in 44 (Figure 3).

Relative frequencies of non-synonymous HA mutations per vaccine status of their associated host, per known risk of antigenic drift – from 0, no knowledge to 3,high known risk-.

**Figure:**
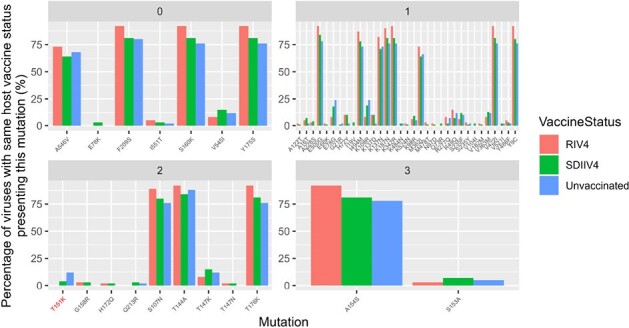

Risk of antigenic drift were the ones identified by FluSurver[3]. Red color of “T151K” tick highlights the fact this is the unique mutation where frequency in vaccinated and in unvaccinated sub-populations is statistically different (p-value=0.023).

**Conclusion:**

Preliminary results revealed an increased frequency of mutations in the HA and NA genes of vaccinated cases relative to non-vaccinated cases. Holistic characterization of influenza viruses can help contextualize the effectiveness of influenza vaccines, especially in a season of substantial mismatch. Further analyses, including with robust sample size, could consolidate these findings, shedding light on the impact of viral mutations on host immune escape.

**Disclosures:**

**Ombeline Jollivet, MSc**, Sanofi: Employee|Sanofi: Stocks/Bonds (Private Company) **Sandra S. Chaves, MD, MSc**, Sanofi: Employee|Sanofi: Employee|Sanofi: Stocks/Bonds (Private Company)|Sanofi: Stocks/Bonds (Private Company) **Christophe Carré, PhD**, Sanofi: Employee **Nicola P. Klein, MD, PhD**, CSL Seqirus: Grant/Research Support|GlaxoSmithKline: Grant/Research Support|Merck: Grant/Research Support|Pfizer: Grant/Research Support|Sanofi Pasteur: Grant/Research Support **Kamela Ng, PhD**, Sanofi: Employee|Sanofi: Stocks/Bonds (Private Company) **Ruvim Izikson, MD, MPH**, Sanofi: Employee|Sanofi: Stocks/Bonds (Private Company) **Heidi Kabler, MD**, Sanofi: Employee|Sanofi: Stocks/Bonds (Private Company) **Clotilde El Guerche-Séblain, PhD**, Sanofi: Employee|Sanofi: Stocks/Bonds (Private Company)

